# Radiotherapy of Breast Cancer—Professional Guideline 1st Central-Eastern European Professional Consensus Statement on Breast Cancer

**DOI:** 10.3389/pore.2022.1610378

**Published:** 2022-06-23

**Authors:** Csaba Polgár, Zsuzsanna Kahán, Olivera Ivanov, Martin Chorváth, Andrea Ligačová, András Csejtei, Gabriella Gábor, László Landherr, László Mangel, Árpád Mayer, János Fodor

**Affiliations:** ^1^ Centre of Radiotherapy, National Institute of Oncology, Budapest, Hungary; ^2^ Department of Oncology, Semmelweis University, Budapest, Hungary; ^3^ Department of Oncotherapy, University of Szeged, Szeged, Hungary; ^4^ Faculty of Medicine, University of Novi Sad, Novi Sad, Serbia; ^5^ Department for Radiation Oncology, Oncology Institute of Vojvodina, Sremska Kamenica, Serbia; ^6^ Department of Radiation Oncology, St. Elisabeth Cancer Institute, Slovak Medical University, Bratislava, Slovakia; ^7^ Department of Oncoradiology, Markusovszky University Teaching Hospital, Szombathely, Hungary; ^8^ Oncoradiology Centre, Bács-Kiskun County Hospital, Kecskemét, Hungary; ^9^ Municipal Oncoradiology Centre, Uzsoki Street Hospital, Budapest, Hungary; ^10^ Oncotherapy Institute, University of Pécs, Pécs, Hungary

**Keywords:** breast cancer, radiotherapy, guidelines, radiation oncology, consensus

## Abstract

The international radiotherapy (RT) expert panel has revised and updated the RT guidelines that were accepted in 2020 at the 4th Hungarian Breast Cancer Consensus Conference, based on new scientific evidence. Radiotherapy after breast-conserving surgery (BCS) is indicated in ductal carcinoma *in situ* (stage 0), as RT decreases the risk of local recurrence (LR) by 50–60%. In early stage (stage I-II) invasive breast cancer RT remains a standard treatment following BCS. However, in elderly (≥70 years) patients with stage I, hormone receptor-positive tumour, hormonal therapy without RT can be considered. Hypofractionated whole breast irradiation (WBI) and for selected cases accelerated partial breast irradiation are validated treatment alternatives to conventional WBI administered for 5 weeks. Following mastectomy, RT significantly decreases the risk of LR and improves overall survival of patients who have 1 to 3 or ≥4 positive axillary lymph nodes. In selected cases of patients with 1 to 2 positive sentinel lymph nodes axillary dissection can be substituted with axillary RT. After neoadjuvant systemic treatment (NST) followed by BCS, WBI is mandatory, while after NST followed by mastectomy, locoregional RT should be given in cases of initial stage III–IV and ypN1 axillary status.

## Introduction

Radiation therapy (RT) remains an essential part of complex breast cancer therapy that according to recent treatment trends are based on both the risk status and use of individualized RT technique chosen also considering the input from the patient. Results published in the past 3 years since the 3rd Breast Cancer Consensus Conference did not bring about fundamental changes in the clinical practice of radiation therapy, but modification and updating the radiation therapy guidelines is necessary based on new scientific evidence. RT after breast-conserving surgery (BCS) is indicated in ductal carcinoma *in situ* (stage 0), since it decreases the risk of local recurrence (LR) by 50–60% (1–9). In early stage (stage I-II) invasive breast cancer RT remains a standard treatment following BCS; however, in elderly (≥70 years) patients with stage I, hormone receptor-positive tumour, the alternative treatment choice of hormonal therapy without RT can be considered (10–17). Hypofractionated (15 × 2.67 Gy) whole breast irradiation (WBI) is a validated alternative that is equivalent to the conventional five-week (25 × 2 Gy) WBI (18–23). In selected cases, RT of the entire breast is not required following BCS, and RT of the tumour bed and surrounding tissues (so-called accelerated partial breast irradiation; APBI) can be used as a substitute (24–36). Following mastectomy, RT significantly decreases the risk of LR and improves overall survival of patients who have 1 to 3 (pN1a) or ≥4 (pN2a, pN3a) positive axillary lymph nodes (37–45). For patients with positive lymph nodes, evidence from a randomized clinical trial supports radical mastectomy followed by hypofractionated RT of the chest wall, axillary apex, and supraclavicular region (21). There is no high-level evidence for the hypofractionated treatment of parasternal lymph nodes. According to the latest randomized trials (EORTC 22922/10925 and NCIC-CTG MA.20), regional RT significantly improves both disease-free and distant metastasis-free survival, while its effects on overall survival are contradictory (46–47). Based on the latest surgical studies, in selected cases with one to two positive sentinel lymph nodes there is no need for complementary axillary dissection. But with the exception of micrometastases (pN1mi) irradiation of axillary lymph nodes or—depending on individual risk—lymph nodes in other nodal regions it is recommended (48–52). In all indications (DCIS, invasive breast cancer and regional irradiation) intensive research is in progress to predict the benefit of RT using various molecular markers with the aim of deescalating therapy in low-risk cases that do not require RT (53).

After neoadjuvant systemic treatment (NST) followed by BCS, WBI is mandatory, while after NST followed by mastectomy, postoperative RT should be given in cases of initial stage III–IV and ≥ypN1 axillary status (54–65). For a great majority of patients, RT is based on high-level evidence. In the future, clinical validation of molecular and genetic markers can provide better personalized RT. The following RT recommendation categories are based on the levels of evidence supporting treatment guidelines and agreement between members of the expert panel:

Evidence levels:

1. Meta-analysis of randomized trials
2. Randomized trials
3. Prospective trial, retrospective studies
4. Expert opinions

Recommendation categories:

1: Full consensus, level 1 evidence
2a: Full consensus, level 2–3 evidence
2b: Generally broad consensus, level 2–3 evidence
3: No consensus, level 2–4, equivocal study results, or few or complete lack of empirical evidence.

## Radiation Therapy Principles—Technical Criteria for Irradiation, Target Volumes, and Dosing

The entire treatment plan must be reviewed before beginning RT. The patient must be informed of the benefits and expected adverse reactions of RT. RT is contraindicated during pregnancy.

The use of three-dimensional conformal RT (3D-CRT) or other up-to-date modalities (IMRT, VMAT, or brachytherapy) are recommended to achieve control of the doses irradiated to the target volumes and the surrounding healthy tissues. Internationally recognized limit values are available for the description of dose coverage and—especially in the case of hypofractionated RT—dose homogeneity as well as the doses received by healthy tissues [heart, LAD, lungs, contralateral breast, or in the case of accelerated partial breast irradiation (APBI) the ipsilateral breast, and in some cases the cervical vessels or brachial plexus].

Healthy organs are protected in multiple ways, for example, when treating the left side, the heart may be protected by deep inspiration and breath-holding as well as individual positioning (prone versus supine). In certain cases the dose received by organs at risk can only be kept at acceptable levels by dose optimisation with inverse treatment planning.

### Whole Breast Irradiation

Target volumes: The whole residual breast. “Boost” treatments delivered with brachytherapy use clinical target volume (CTV) specifying the area corresponding to the original tumour with a 2 cm safety zone (RT safety zone = 20 mm—intact surgical margin in mm) (recommendation category: 2b) (66). Hence, in brachytherapy, no additional PTV-CTV expansion is necessary (PTV=CTV). In teletherapy, the recommendation is to extend the CTV by an additional 0.5 cm when using fractionated image-guided RT and an additional 1 cm when performed without image guidance; the latter is to compensate for the greater inaccuracy of the settings and displacement due to respiration (PTV) (28). The size of PTV-CTV extension requires individual consideration depending on the patient fixation and QA protocols used in each centre. If the surgical margin cannot be defined in each direction, then “boost” irradiation is to be performed to the tumour bed plus a 15 mm extension (CTV) (recommendation category: 3).

Technical criteria for optimal radiation therapy: Megavoltage irradiation (4–10 MV photon), CT-based three-dimensional conformal radiation therapy (3D-CRT), and use of tangential fields. The exposure of the lungs and the heart must be minimised (recommendation category: 1) (67–71), which in a certain proportion of cases will require the use of special irradiation techniques (irradiation during deep inspiration and breath-holding or respiratory gating, intensity-modulated RT—IMRT, or RT with the patient in prone position) (70–79). Simple control measurements include the central lung distance (<3 cm) and the maximal heart distance (<1 cm), and a more accurate method is the analysis of the dose-volume histogram (DVH). In order to achieve homogeneous dose distribution, the target volume can be divided into subfields (segments) or compensators and wedge fields can be used, while IMRT is recommended for large breasts (recommendation category: 2a) (80). Additional (“boost”) dose can be directed to the tumour bed with an accelerator (electron, photon) or with brachytherapy (usually interstitial needle implant). For small breasts and more superficial tumours, electron beam irradiation is usually preferable; for large breasts and deeply situated tumours brachytherapy or 3D conformal photon “boost” is recommended. When using “boost” treatment, the tumour bed must be intra-operatively marked with titanium clips to avoid the geographical miss of the target volume (67, 81–83). For oncoplastic procedures, appropriate documentation and communication about the surgery are important.

Ideally, RT should start after wound healing, the recommended period is 4–6 weeks—but no more than 12 weeks—after the surgery (recommendation category: 2a). Following adjuvant chemotherapy, RT is started after a 3-week off-treatment period after the last chemotherapy cycle (recommendation category: 2b). Accelerated partial breast irradiation (APBI) may also be administered prior to the adjuvant chemotherapy (15). Percutaneous “boost” treatment is performed after whole breast irradiation and without a break—as long as no radiation dermatitis of more than grade 2 is present (recommendation category: 2b). In case of serious (grade 3) dermatitis a 1–3 week-long off-irradiation period can be considered. While brachytherapy “boost” treatment is usually performed 1–3 weeks after the completion of whole breast irradiation, it can also be performed as a perioperative brachytherapy “boost” therapy before the whole breast irradiation (recommendation category: 3). Percutaneous “boost” therapy can be combined with IMRT as a simultaneous integrated “boost” treatment (SIB) (recommendation category: 2b).

Dosing: The basic dose schedule is 40–42.5 Gy (2.67 Gy/fraction, 5 times a week, in 15–16 fractions) or 45–50.4 Gy (1.8–2 Gy/fraction, 5 times a week, in 25–28 fractions) (recommendation category: 2a). Dose usually refers to that given to the isocentre. Accelerated hypofractionated treatment requires paying close attention to the dose limits of organs at risks (heart and lungs) and to dose homogeneity, which provides local tumour-free results identical to standard fractionation and the fractionation schemes are also at least equivalent in terms of adverse reactions (18–23). Based on the 5-year results of the FAST-FORWARD trial extreme hypofractionation (e.g., 5 fractions of 5.2 Gy on 5 consecutive days) is a promising treatment option. However, longer follow-up and further prospective trial data are needed before its implementation into the routine daily practice. Extremely hypofractionated WBI should be considered only in the context of prospective clinical trials. Additional (“boost”) dose of the tumour bed is performed with external RT of 10–16 Gy (2 Gy/fraction, 5 times a week), or with high dose rate (HDR) brachytherapy 1 × 10 Gy or 3×4–5 Gy. SIB IMRT includes 50 Gy (2 Gy/fraction, 5 times a week) irradiation to the whole breast and 60 Gy (2.4 Gy/fraction, 5 times a week) to the “boost” target volume, or using hypofractionated treatment with 21 × 2.17 Gy to the whole breast +21 × 2.66 Gy to the “boost” target volume (total doses received by the whole breast and tumour bed are 45.6 Gy and 55.9 Gy, respectively), but many other variations of fractionation can be used.

### Accelerated Partial Breast Irradiation

Target volume: Determination of target volume is based on tumour bed markers (clips) (67, 84). In the absence of tumour bed markers, US or CT can be used to determine the target volume (recommendation category: 2b). In the absence of clips, partial breast irradiation is only feasible if the tumour bed is clearly visible and identifiable using imaging methods (“cavity visibility score”; CVS≥3) (85). The clinical target volume (CTV) for brachytherapy is the area corresponding to the original tumour with a 2 cm safety zone (RT safety zone = 20 mm—intact surgical margin in mm) (recommendation category: 2b) (67). In brachytherapy, no additional PTV-CTV expansion is necessary (PTV=CTV). In teletherapy, the recommendation is to extend the CTV by an additional 0.5 cm when using fractionated image-guided RT and an additional 1 cm when performed without image guidance; the latter is to compensate for the greater inaccuracy of the settings and displacement due to respiration (28). The size of PTV-CTV extension requires individual consideration, depending on the patient fixation and QA protocols used in each centre. When administering partial breast irradiation with teletherapy, fractionated image guidance is recommended to decrease target volume extension and to minimize adverse reactions (recommendation category: 2b).

Technical criteria for optimal radiation therapy: APBI can be administered via interstitial brachytherapy, 3D-CRT or IMRT (24–31, 33–36, 86). Partial breast irradiation using a single high-dose (1×20–21 Gy) intraoperative electron beam therapy or low energy (50 kV) X-ray therapy significantly increases the risk of local recurrence and therefore cannot be recommended for routine care (recommendation category: 2a) (87, 88). Accelerated hypofractionated treatment requires paying close attention to the dose limits of organs at risk (heart and lungs).

Ideally, RT should start after wound healing, and the recommended period is 4–6 weeks—but no more than 12 weeks—after the surgery (recommendation category: 2a). After adjuvant chemotherapy, RT is started at the end of a 3 weeks off-treatment period after the last chemotherapy cycle, but due to its short treatment period (4–5 days) accelerated RT can also be administered before chemotherapy without the risk of a significant delay in systemic treatment (recommendation category: 2b).

Dosing: Fractionated HDR or ultrafractionated PDR afterloading technique. Using PDR brachytherapy, the dose is 45–50 Gy, and the dose rate is <1 Gy/hour. Fractionated HDR brachytherapy of 7 × 4.3 Gy, 8 × 4 Gy, or 10 × 3.4 Gy, two daily treatments (leaving at least 6 h between the fractions) (29, 30, 32–34). At present APBI with extreme hypofractionation (1–4 treatment fractions, in 1–2 days) can only be administered in prospective clinical studies (25, 26, 35). Appropriate dose homogeneity (dose homogeneity index; DHI>0.65) and at least 90% coverage of target volume (coverage index; CI ≥ 0.9), maximum skin dose <70%. Using 3D-CRT or IMRT the dose is 9 × 4.1 Gy or 10 × 3.85 Gy, with two daily treatments (27, 28, 34, 36), or 5 × 6 Gy or 15 × 2.67 Gy, with one daily treatment (24). Dose prescription for ICRU point (isocentre; 100%). PTV coverage: V95_PTV_=100% (PTV is covered by the 95% isodose surface).

### Chest Wall Irradiation

Target volume: Operated chest wall area with surgical scar and lobe, and if possible, the site of the surgical drain.

Technical criteria of optimal radiation therapy: Use of up-to-date megavoltage machine (high-energy photon or electron beam), CT-based RT planning, at least 3D-CRT recommended to provide maximum protection for the heart and lungs. Use of tangential photon or direct electron field(s). In order to achieve even dose distribution, subfields (segments), compensators (wedge filters), or bolus or IMRT can be used.

Ideally, RT should start after wound healing, and the recommended period is 4–6 weeks—but not more than 12 weeks—after the surgery (recommendation category: 2a). When administering adjuvant chemotherapy, RT is started after a 3 week off-treatment period following the last chemotherapy cycle (recommendation category: 2b).

Dosing: The standard dose schedule is 40–42.5 Gy (2.66–2.67 Gy/fraction, 5 times a week) or 45–50.4 Gy (1.8–2 Gy/fraction, 5 times a week). In the START studies (START-A, B, and Pilot) less than 10% of the patients (513 patients out of 5,861) were treated with mastectomy (23). The 5-year results of the Chinese post-mastectomy randomized study were published in 2019 (21). The rates of locoregional results and complications were similar with hypofractionation (15 × 2.9 Gy) and conventional fractionation (25 × 2 Gy). The parasternal lymph nodes were not irradiated (recommendation category: 2a) (19–23). Extremely hypofractionated WBI should be considered only in the context of prospective clinical trials. Accelerated hypofractionated treatment requires paying close attention to the dose limits of organs at risk (heart and lungs) and dose homogeneity. If there is a positive or close (<2 mm) surgical margin a “boost” dose applied to the surgical scar is 10–16 Gy (2 Gy/fraction, 5 times a week) (89).

### Irradiation of the Lymphatic Regions

The definition of the target volume depends on the type of axillary surgery (sentinel lymph node biopsy or axillary dissection) and the pathology status of the axillary lymph nodes. When performing sentinel lymph node biopsy, it is recommended that a surgical clip be placed at the site of the excised lymph node (recommendation category: 2b). This marker can assist in the assessment of dose coverage in various field layouts and irradiation techniques (66). When the patient is in a supine position during RT, the exposure of the lymphatic regions from tangential fields is inadequate (90), and the use of fitted additional fields has not been widely used due to the uncertainty originating from repositioning.

Levels 1–3 of the axilla for the contouring of the medial supraclavicular and parasternal lymph node regions must follow the anatomical borders (90–94). According to the newest European recommendations, exposure of the cervical vessels must be avoided during irradiation of the medial supraclavicular region (93).

#### Target Volumes

– Level I of the axilla
– Level II of the axilla (frequently treated together with the interpectoral/Rotter lymph nodes)
– Level III of the axilla
– Medial supraclavicular region (also called level IV of the axilla)
– Parasternal/internal mammary region

#### Conventional Field Layouts

– Elective supraclavicular field: supraclavicular and infraclavicular region + axillary apex (levels III–IV)
– Supraclavicular-axillary field: supraclavicular and infraclavicular region + axilla (levels I–IV or levels II–IV)
– Parasternal field: ipsilateral parasternal area including at least the first three intercostal spaces.

#### Technical Criteria of Optimal Radiation Therapy

– Supraclavicular-axillary region: Megavoltage irradiation (4–10 MV photon) with 3D conformal RT planning. During concurrent irradiation to the nodal region and the residual breast or the chest wall, the use of a single isocentre or the IMRT technique produce the best dose distribution.
– Parasternal region: The position of the parasternal lymph nodes (target volume: ipsilateral intercostal spaces 1–4) is indicated by the course of the internal mammary artery. Use of deep tangential fields should be avoided since the exposure of the critical organs (heart and lungs) is significant under such circumstances. Use of 3D-CRT or IMRT is essential to decrease the radiation exposure of the heart and lungs, and in some cases compromising the dose administered to the parasternal lymph nodes (46–48 Gy) may be necessary.
– Dosing: 40 Gy (2.67 Gy/fraction, 5 times a week) or 45–50.4 Gy (1.8–2 Gy/fraction, 5 times a week) (recommendation category: 2a) (18–23). Hypofractionation is still controversial when used for the irradiation of the internal mammary chain of lymph nodes, and some experts recommend conventional fractionation (recommendation category: 3).

### Radiation Therapy of the Locoregionally Advanced Breast Cancer

Target volumes: Breast or chest wall, all unilateral lymphatic regions, and for “boost” treatments the tumour or tumour bed +1.5–2 cm safety zone.

Technical criteria of optimal radiation therapy: see the previous three chapters.

Dosing: The standard dose for target volumes is 50 Gy (2 Gy/fraction, 5 times a week), since in most cases the internal mammary chain is part of the target volume (recommendation category: 2b). Hypofractionated application of the dose (40–42.5 Gy in 15 fractions) in locoregionally advanced breast cancer is not supported by high-level evidence, but it can be used, based on positive experiences in prospective studies following breast-conserving surgery (recommendation category: 3). Additional (“boost”) irradiation of 10–26 Gy is recommended for residual tumours.

## Radiation Therapy Treatment Guidelines

### *In situ* Breast Cancer (Stage 0, pTis N0 M0)

#### Lobular Carcinoma *In situ*

RT is not necessary (recommendation category: 2a).

#### Ductal Carcinoma *In situ*

• Irradiation is usually recommended after breast-conserving surgery, because 50 Gy administered to the residual breast decreases the risk of local recurrence by 50%–60% in all risk groups (recommendation category: 1) (1–9). Usually, 50% of local relapses are DCIS and the other 50% are invasive cancer. For low-risk patients (well-differentiated lesion, with minimal or no necrosis, at least 10 mm safety zone, >60 years of age) radiation therapy may be omitted based on individual assessment (recommendation category: 3). The significance of “boost” dose is still unclear. For young (≤45 years of age) patients (recommendation category: 2a) or high-grade DCIS or narrow surgical margin (<2 mm) (recommendation category: 3) a boost dose should be considered (8, 95). Partial breast irradiation for DCIS can only be administered in the framework of a prospective clinical trial (recommendation category: 3) (31).
• Chest wall RT is not required after mastectomy (recommendation category: 2A).
• Irradiation of lymphatic regions is not justified: pTis N0 M0 (recommendation category: 2A).
• In cases of Paget’s disease of the nipple, wide cone excision should be followed by RT of the residual breast (recommendation category: 1).

### “Early Stage” Invasive Breast Cancer: Stage I−II, T1-2 N0-1 M0, T3 N0 M0

#### Partial Mastectomy Followed by Irradiation of the Residual Breast Tissue

Contraindications of breast-conserving surgery (in cases of DCIS and of invasive cancer):

– Prior RT of the breast or chest wall
– Pregnancy—if the postoperative RT would be administered during the pregnancy
– Diffuse microcalcification (suggestive of malignancy)
– Positive surgical margin after reexcision
– Connective tissue disease: scleroderma, lupus (relative contraindication)
– Patient is germline TP53- and ATM-mutation carrier
– Premenopause with known BRCA1-2 mutation (relative contraindication), due to a high risk of local recurrence (second primary tumour). Breast-conserving surgery requires prior discussion with the patient with detailed description of future risk of cancer.
• Irradiation of the residual breast decreases the risk of local recurrence by 75% in all age groups (recommendation category: 1) (10, 11, 14–17). RT also significantly improves the 15-year breast cancer-specific survival—by 5% and 7% in patients with negative or positive lymph nodes, respectively (10). Accelerated hypofractionated whole breast irradiation (15 × 2.67 Gy or 16 × 2.66 Gy) is an equivalent alternative to conventional fractionation (50 Gy/5 weeks), provides local tumour-free results identical to standard fractionation and does not increase the incidence or severity of late adverse reactions (recommendation category: 1) (18–23). Based on the first results from the FAST-Forward randomized study, whole breast irradiation of 5 × 5.2 Gy administered over 1 week is effective, and at the 5-year follow-up point does not increase the rate of late adverse reactions. However, taking into consideration the scarcity of experience and lack of long-term follow-up results, its use outside of the clinical study setting is currently not recommended (recommendation category: 3) (96). In young patients whole breast RT may be used after chemotherapy and with concurrent regional RT and “boost” irradiation (recommendation category: 2b) (18–21, 23). For older (≥70 years of age) patients with good prognosis (stage I, negative surgical margin, hormone receptor-positive tumour) discontinuing RT and using only endocrine therapy can be considered—with the informed consent of the patient—since RT does not improve 10-year overall survival, but the patient must be fully informed of the significantly higher risk of local recurrence (at 10 years the rate is 10% without RT and 2% with RT) and its consequences (recommendation category: 2a) (12, 13).
• Treatment of the tumour bed with elevated (“boost”) dose improves local tumour control in all risk groups, but for low-risk patients the absolute benefit of this treatment is limited (≤3% at 20-year follow-up) (recommendation category: 1) (15, 81–83, 97, 98).

#### Indications of an Additional (“Boost”) Dose

Absolute indication (the presence of just one of the conditions is sufficient) (recommendation category: 1):

– Microscopically positive surgical margin (in the absence of reexcision)
– Small surgical margin (intact surgical margin <2 mm)
– ≤50 years of age
– Triple-negative breast cancer
– Poorly-differentiated (grade 3) tumour

Relative indication (recommendation category: 2A):

– Extensive intraductal component (EIC)
– Lymphovascular invasion
– Mitotic activity index (MAI) > 10 (/10 NNL)
– pT ≥ 3 cm
• Accelerated partial breast irradiation (APBI) is the standard treatment alternative to whole breast irradiation in selected low-risk cases (32). When adjuvant chemotherapy is indicated, APBI may be administered either before chemotherapy or after the completion of chemotherapy (15). Using interstitial brachytherapy with appropriate technique or external RT (3D-CRT or image-guided IMRT) the local tumour-free results are non-inferior compared to those achieved with whole breast irradiation, and the rate of late adverse reactions is not higher (recommendation category: 2a) (24, 27–34). For patients who prefer APBI with 3D-CRT or IMRT, once daily fractionation (15 × 2.67 Gy over 3 weeks) should be chosen (24), or if twice-daily fractionation (9 × 4.1 Gy or 10 × 3.85 Gy) is preferred then the patient should be informed of the potential benefits and risks of external RT as well as of the fact that contradictory results have been published regarding the cosmetic results and late adverse reactions of APBI with twice-daily fractionation (28, 34, 36, 86) (recommendation category: 2B). When using twice-daily fractionation, the target volume should be kept below 160 cm^3^ (28, 34) (recommendation category: 2b).

#### Indications of APBI

Low-risk patients who are eligible for APBI outside of the framework of a clinical trial (recommendation category: 2a) (31):

– >50 years of age and
– Unicentric/unifocal invasive carcinoma and
– pT1-2 (≤30 mm) tumour size and
– Negative surgical margin and
– pN0 axillary status (with sentinel lymph node biopsy or axillary dissection) and
– EIC-negative tumour and
– Absence of lymphovascular invasion

Note: All criteria must be simultaneously met.

Moderate-risk patients are only eligible for APBI in the framework of a prospective clinical trial or after obtaining informed consent. If APBI is used outside of a clinical trial, the patient must be informed about the paucity of long-term results and what that means in terms of potential risks (recommendation category: 3) (31):

– >40–50 years of age or
– Unicentric, but multifocal tumour (within 2 cm of the primary tumour) or
– Pure DCIS or
– pN1mi (micrometastasis)

Note: The presence of only one criterion is sufficient to meet the moderate-risk status.

High-risk patients for whom APBI is contraindicated (recommendation category: 1) (31):

– ≤40 years of age
– pT2 (>30 mm), pT3, pT4 tumour size
– Positive surgical margin
– Multicentric or multifocal tumour (spreading beyond 2 cm of the primary tumour)
– EIC-positive tumour
– Positive for lymphovascular invasion
– pNx (unknown) or pN1a-2a-3a [1 or more macroscopic (>2 mm) positive lymph node] axillary status
– Breast-conserving surgery after prior neoadjuvant chemotherapy

Note: The presence of only one of the criteria is sufficient to meet this risk category.

#### Chest Wall Irradiation After Mastectomy

• pT1-2 pN0-1mi: Irradiation is not needed if the tumour was resected with intact surgical margins (recommendation category: 1). Although chest wall irradiation slightly decreases the rate of local recurrence at 5 years (from 1.9% to 1.2%), it does not improve breast cancer-specific survival (37). According to the NCCN protocol, chest wall irradiation should be considered if the intact surgical margin is ≤ 1 mm (15).
• pT3 pN0: Chest wall irradiation is recommended (recommendation category: 2a) (38).
• pT1-2 pN1a-2a-3a: Locoregional RT is recommended (recommendation category: 1).
• RT decreases the incidence of local recurrences at 5 years by ∼15% (1–3 positive lymph nodes: from 17% to 3%, 4 or more positive lymph nodes: from 26% to 11%) and improves 20-year breast cancer-specific survival by 8–10% (37).
• pT1-2 pNx or pN0 but <6 examined lymph nodes (except when sentinel lymph node biopsy was performed): irradiation should be considered (recommendation category: 2b).
• Immediate breast reconstruction following mastectomy: the reconstructed breast and the chest wall are treated according to the above guidelines. The two-stage procedure provides a better result than immediate reconstruction with implant: expander insertion, irradiation of the expander, and after the irradiation the expander is replaced with the permanent implant.

#### Sentinel Lymph Node Biopsy Followed by Irradiation of the Axillary-Supraclavicular Region

• pN0-1mi (sn): If the sentinel node (SN) is negative or if there is a micrometastasis, usually there is no need for nodal irradiation (recommendation category: 2a), but irradiation of axilla levels 1–2 should be considered if there is an increased risk (histology indicated an aggressive tumour, >pT1, multifocality, presence of LVI, a single SN was removed, systemic therapy is anticipated to have low or no efficacy, young age of the patient) (recommendation category: 3).
• pN1a (sn): If there is a macrometastasis (>2 mm) in the sentinel lymph node and axillary dissection is performed, then RT of the supraclavicular region (level 4) and axillary apex (level 3) is recommended, while there is no need to irradiate levels 1–2 (recommendation category: 2a). If no axillary dissection is performed (according to ACOSOG Z011 criteria), then irradiation of the axillary lymph nodes and, based on individual risk, other regional lymph nodes is required, since an incidence of metastases of 27–38% is estimated in the non-dissected non-sentinel lymph nodes (recommendation category: 2b) (48, 50, 99). Generally, levels 1–4 of the axilla are irradiated (recommendation category: 2a), but in the presence of lower risk it is sufficient to irradiate levels 1–2 of the axilla (favourable histology, pT1, unifocality, only one of several sentinel lymph nodes is involved, size of macrometastasis is <7 mm, effective systemic therapy, relatively older patient; recommendation category: 3). The RT performed instead of axillary dissection is equivalent to surgery in terms of nodal relapse-free results and overall survival (recommendation category: 1) (51, 52).

#### Axillary Lymphadenectomy Followed by Irradiation of the Axillary-Supraclavicular Region

• pN0-1mi: RT is not necessary (recommendation category: 1).
• pN1a, 2a, 3a, pN3c (ipsilateral sub-/supraclavicular lymph node metastasis): RT of the supraclavicular region and axillary apex is recommended (recommendation category: 2a) (46, 47, 100). If there has been adequate axillary dissection (≥6 removed lymph nodes) the use of elective supraclavicular field is sufficient, while irradiation of levels 1–2 is not required (recommendation category: 2a).
• pNx or pN0 but <6 examined lymph nodes (except when sentinel lymph node biopsy was performed): After inadequate lymphadenectomy (<6 processed lymph nodes) RT of the supraclavicular and axillary region (levels 2–3) is recommended based on individual consideration, but in general the targeted irradiation of axillary level 1 is not required (recommendation category: 2b).

#### RT of Lymph Nodes Along the Internal Mammary Artery

• pN0-1mi: Irradiation is not necessary (recommendation category: 2a).
• pN1a, pN2a, pN3a: If there are four or more positive axillary lymph nodes, RT of the parasternal region is recommended; if there are 1–3 positive lymph nodes, the decision to use RT is to be based on the individual consideration of the organs at risk doses and on the benefit gained represented by the risk of parasternal lymph node metastasis (recommendation category: 2b) (46, 100, 101). The value of elective irradiation of parasternal lymph nodes has not been elucidated completely, and radiation therapy professionals should always consider the risks of lung and heart exposure. Clinical manifestation of parasternal lymph node recurrence is very rare (<1%) and according to the latest published studies the role of parasternal lymph node irradiation in improving overall survival is not fully clarified, hence the routine elective RT of this region is still controversial (recommendation category: 3) (46, 100, 101).
• pN1b, pN1c, pN2b, pN3b: If there is histologically confirmed internal mammary sentinel lymph node or clinically unequivocal (CT, UH, MRI) parasternal lymph node metastasis, irradiation is recommended even in the presence of negative axillary status (recommendation category: 2a).

### Radiation Therapy After Neoadjuvant Systemic Therapy

The efficacy of individualized neoadjuvant systemic therapies continues to improve, as shown by the improvement in pCR rates. A good response indicates a lowered risk of locoregional relapse, and in some cases RT can be avoided. A key consideration is that neoadjuvant systemic therapy can reduce the need for radical RT, which is one of its anticipated benefits compared to the adjuvant scheme.

#### Radiation Therapy After Neoadjuvant Systemic Therapy and Breast-Conserving Surgery

RT of the residual breast is recommended in all cases (recommendation category: 1) (54, 55, 64). After administration of the 50 Gy standard dose, a 10–16 Gy tumour bed “boost” should be considered (recommendation category: 3). The use of moderately hypofractionated regimens (e.g., 15 × 2.67 Gy or 16 × 2.66 Gy) for whole-breast irradiation are also acceptable. Administration of this additional dose should be based on the usual risk factors (age, histological type, initial grade, multifocality, surgical margin, lymph node status, and the detection of vessel invasion). In the NSABP B-18 and B-27 trials, the rate of local recurrence among patients receiving breast-conserving surgery and breast-only irradiation was around 10% (64). The predictors of local recurrence are the following: lack of pCR (especially ypN positivity), young age (<50 years), and advanced initial stage. In a similar patient group treated at the MD Anderson Cancer Center, in addition to advanced initial grade the following factors proved to be predictors of local recurrence: cancer of grade 3, and hormone receptor-negativity, presence of lymphovascular invasion, multifocal residual carcinoma, and close surgical margin (54, 56).

#### Chest Wall Irradiation After Neoadjuvant Systemic Therapy and Mastectomy

Clinical stage II: If there is a negative surgical margin (and ypN0 axillary status) RT is not required (recommendation category: 2b) (54, 55, 57–63, 65). If there is a positive surgical margin 50 Gy chest wall irradiation +10 Gy boost (2 Gy/day) irradiation is recommended and should be administered in all cases in which irradiation of the lymphatic regions is necessary (recommendation category: 2a).

Clinical stage III−IV: In case of negative surgical margin (and ypN0 axillary status) 50 Gy chest wall irradiation, in case of positive surgical margin 50 Gy chest wall irradiation +10 Gy boost (2 Gy/day) irradiation is generally recommended and should be administered in all cases in which irradiation of the lymphatic regions is necessary (recommendation category: 2a) (54, 55, 57–63, 65). With the improving efficacy of systemic treatments, we can anticipate that pCR cases with no need of RT will become increasingly common, provided that the initial stage of the tumour is not advanced. Mastitis carcinomatosa cases require a wide (≥10 mm) safety margin during RT.

#### Irradiation of the Lymphatic Regions

At present, recommendations for optimal surgical and RT care following neoadjuvant systemic therapy are still based on retrospective data (101, 102). According to the Sentina clinical study, 70% of sentinel lymph node-positive cases become sentinel lymph node-negative in response to neoadjuvant chemotherapy, and in such cases regional irradiation can be omitted. At the same time, data published on the use of old diagnostic and therapeutic options suggest that regional irradiation is expected to have significant benefit in cases with initially advanced tumor stage and/or lymph node status (cN2-3) (53–55). A recent publication based on the analysis of a large database has highlighted the significance of the pathological tumor response in predicting the benefit of regional irradiation (102). Accordingly, in cases with initially positive lymph node status achieving ypN0 in response to neoadjuvant therapy, the benefit of irradiation is primarily associated with the histological type (hormone receptor-negativity) rather than the initial lymph node status. In such cases the indication of regional irradiation should be determined on an individual basis. The results of the first randomized trial (NSABPB-51/RTOG 1304) on regional irradiation used in cases with cN1→ypN0 are expected to be published in 2020 (103-108).

Table 1 shows the recommendations on regional irradiation following neoadjuvant systemic therapy (recommendation category: 2b) (54, 55, 57–63, 65).

**TABLE 1:** Recommendation for regional irradiation after neoadjuvant systemic therapy.

Initial status	Status after neoadjuvant treatment	Surgical (pathology) status	RT indication
cN0	cN0	ypN0 (sn)	No RT
cN0	cN0	ypN1 (sn)	ABD ± RT or RT
cN1 (f), pN1 (sn)	cN0-1	ypN0	No RT
cN1, pN1 (sn)	cN1	ypN1-2–3	RT
cN2 (f)	cN0	ypN0	RT or OBS

### Radiation Therapy After Breast Reconstruction

#### Reconstruction With Silicone Implant

Irradiation can be administered with no significant change in dosing. Nevertheless, an elevated risk of capsular contracture must be considered. Good cosmetic results can be achieved with careful fractionation and moderate dose (45–50.4 Gy, 1.8 Gy/fraction), and eschewing a bolus or additional dose (recommendation category: 2b) (109, 110). Hypofractionated treatment schedules are not recommended in the case of silicone implants, but in case of implanting a temporary expander by a two-stage reconstruction procedure, hypofractionated doses may be given.

#### Reconstruction With Autologous Tissue

RT does not significantly compromise the cosmetic results. The restrictions regarding silicone implants do not apply here (recommendation category: 2b) (109, 110). Use of hypofractionated treatment schedules is not recommended.

### Irradiation and Systemic Treatment

• RT is administered after chemotherapy (recommendation category: 2b), but should be completed within 7 months after surgery (111, 112).
• There is no need to suspend trastuzumab therapy during irradiation (recommendation category: 2b) (111, 113).
• Aromatase inhibitors can be administered concurrently with RT (recommendation category: 2b) (111, 112, 114). Concurrent administration of tamoxifen and RT may increase the rate of grade 1 lung and breast fibrosis, but since its clinical relevance is not proven, concurrent administration can be considered on an individual basis (recommendation category: 3) (111, 112, 114, 115).

### Radiation Therapy for Rare Diseases

#### Occult Breast Cancer (T0 N1-2 M0)

Occult breast cancer (axillary lymph node metastasis without identified primary tumour) requires the removal of axillary lymph nodes. Usually systemic therapy precedes RT (15).

Mastectomy is followed by irradiation of the axillary-supraclavicular region; in the case of breast-conserving surgery both the breast and the axillary-supraclavicular region require irradiation (recommendation category: 2b) (116). The dose is 50 Gy, and an additional 10 Gy “boost” can be applied in the presence of extensive axillary metastasis. Hypofractionated application of the standard dose (40–42.5 Gy in 15 fractions) in occult breast cancer is not supported by high-level evidence, but it can be used based on positive experiences in prospective studies following breast-conserving surgery (recommendation category: 3).

#### Malignant Phyllodes Tumours

Malignant phyllodes tumours are characterized by fast local expansion and high rates of local recurrence. The incidence of axillary metastasis is low (1.5%). The literature data are contradictory with respect to the value of postoperative RT. RT generally decreases the risk of local recurrence, but does not improve tumour-specific survival (117) (recommendation category: 2b). Irradiation with 50 Gy dose is recommended after mastectomy and positive or unclear surgical margin, or for the treatment of residual breast after excision. After breast-conserving surgery, following the standard dose of whole breast irradiation, a dose of 10–16 Gy “boost” should be considered (recommendation category: 3) (117). Borderline tumours require individual consideration.

#### Primary Breast Sarcomas

After breast-conserving surgery, doses of up to 50 Gy are recommended for the residual breast and 60–66 Gy to the tumour bed (recommendation category: 2b) (118). In the case of carcinosarcoma—with positive axillary lymph nodes—RT of the lymphatic regions is also recommended.

#### Secondary Sarcoma (Angiosarcoma) in the Conserved Breast

After mastectomy, reirradiation with hyperfractionated RT on an individual basis can be applied to the chest wall: twice-daily 1.5 Gy (2 fractions per day, at least 6 h between the fractions) with a total dose of up to 60 Gy (recommendation category: 3) (119, 120).

#### Breast Fibromatosis

The treatment is primarily surgical. If radical excision of the lesion is not possible (R1 or R2 resection), postoperative or definitive RT with 50–60 Gy dose can be administered (recommendation category: 2b) (121).

#### Male Breast Cancer

This is an uncommon condition (ratio in men:women is 1:100–200). Evidences for female breast cancer is to be followed during treatment. For patients with operable breast cancer the primary treatment consists of radical mastectomy and removal of axillary lymph nodes (axillary block dissection/sentinel lymph node biopsy). The RT of the chest wall surgical area and the regional lymph nodes is identical to that recommended for female breast cancer cases. Breast-conserving surgery is rarely performed in men. The residual breast is irradiated after breast-conserving surgery (15).

### Radiation Therapy for Locoregional Recurrences

#### Recurrence in the Ipsilateral Breast

• In the absence of prior RT, “salvage” mastectomy (standard treatment) is followed by postoperative RT according to the recommendations for primary treatment (“Chest wall irradiation after mastectomy”) (recommendation category: 2a) (122). After administration of the full dose of RT, repeat irradiation with 40 Gy dose can be carried out (recommendation category: 2b).
• In the absence of prior RT, a second breast-conserving surgery is followed by postoperative RT according to the recommendations for primary treatment (see the first two paragraphs under “Chest wall irradiation after mastectomy”) (recommendation category: 2a). After a prior full dose RT, repeated RT with perioperative interstitial brachytherapy or 3D conformal external RT can decrease the risk of a second local recurrence (recommendation category: 2b) (123–128). Doses: with HDR brachytherapy 22–36 Gy (in 5–10 fractions) (124, 126, 128), with PDR brachytherapy 45–50 Gy (124), or with teletherapy 45 Gy over 15 days (with a 2 × 1.5 Gy/day fractionation schedule) (123).

#### Chest Wall Recurrence

• If no adjuvant irradiation was administered after the first surgery, the entire ipsilateral chest wall must be irradiated (recommendation category: 2b) (122). Use of small fields is not recommended, since in the case of additional recurrences this leads to field adjustment issues and overdosing is unavoidable. The total dose is 50–54 Gy and the usual fractionation (2 Gy/day) is applied. After excision, the scar can be treated with an additional “boost” of 5 × 2 Gy. Extensive, contiguous recurrences are treated with palliative irradiation.
• If adjuvant irradiation was administered after the first surgery, the possibility of repeat irradiation is limited, since overdosing could lead to tissue necrosis or radiation ulcer. Palliative repeat irradiation is performed with individual dosing (usually 30–40 Gy) dependent on the dose of prior RT, using small fields without close fitting (recommendation category: 3) (122). Repeat irradiation using the CORT (Combined Operative and Radiotherapeutic Treatment) technique or HDR brachytherapy can be carried out to a maximal dose of 30 Gy (recommendation category: 3) (127).

#### Axillary Nodal Recurrence

• If no prior irradiation was used, a dose of 50–60 Gy with conventional fractionation (2 Gy/day) is recommended (recommendation category: 2b).
• In the event of prior irradiation, small-volume palliative irradiation is performed and individual dosing dependent on the dose of prior RT (usually 20–30 Gy) (recommendation category: 3).

#### Supraclavicular Metastasis (Recurrence)

• In the absence of prior RT, the entire region is irradiated with up to 50 Gy with conventional fractionation (2 Gy/day). The residual tumour can be treated with an additional “boost” of 5 × 2 Gy (recommendation category: 2B).
• Following a prior RT, a reirradiation dose of up to 30 Gy may be administered for palliative purposes (recommendation category: 3).

### Radiation Therapy of Distant Metastases (Stage IV)

When administering palliative RT, the irradiated target volume, applied total dose and fractionation are less amenable to standardization than the same parameters in curative treatments. Individually tailored treatment is carried out, taking into consideration the extent of the disease, the life expectancy and general condition of the patient, and the dominant symptoms. In general, smaller total dose, single larger fractions (hypofractionation) and simpler irradiation methods are used, but when administering larger doses for palliative purposes, CT-based (if possible, 3D conformal) RT planning is recommended for the sake of healthy tissue protection.

For extracranial solitary metastases or low-volume oligometastatic disease with more favourable expected disease course (e.g., adrenal gland, bone or liver metastases) extracranial stereotactic RT may be an alternative to surgical treatment (metastasectomy).

#### Bone Metastases

• Solitary metastasis: usually 10 × 3 Gy or 5 × 4 Gy, over 1–2 weeks, possibly 1 × 8 Gy (recommendation category: 2a) (121).
• Multiple metastases: the purpose of irradiation is pain reduction and improvement of mobility; in such cases short treatment times are recommended (1×6–8 Gy, 2×4–5 Gy, 5×3–4 Gy, etc., open-field irradiation depending on the extent of the process and the size of the field) (recommendation category: 2a) (129).

Another alternative for the palliative treatment of multiple bone metastases is the use of open radioactive isotopes (strontium-89 chloride, yttrium-90 EDTMP, etc.) (recommendation category: 2a).

#### Brain Metastases

In cases with solitary brain metastasis or oligometastatic disease (2–4 foci) the recommended treatment is stereotactic radiosurgery (SRS) using a single fraction of 15–20 Gy dose or fractionated stereotactic RT (FSRT) in 3 to 5 fractions of 5–9 Gy without irradiation of the entire cranium (recommendation category: 2a), since irradiation of the entire cranium does not improve survival but decreases cognitive function and quality of life (130). Later on, eventual new solitary brain recurrences can be treated with stereotactic RT again by taking into consideration the previously treated target volumes and doses. For multiple brain metastases (>4) or brain metastases unsuitable for stereotactic treatment, whole brain irradiation is recommended. 10 × 3 Gy is sufficient to alleviate symptoms (recommendation category: 2a). For cases with a more favourable prognosis and patients in better general condition, 20 × 2 Gy can be administered to the whole brain, followed by a 5 × 2 Gy “boost” to the affected area using CT-based 3D conformal treatment planning (recommendation category: 2a) (129).

#### Mediastinal Metastasis

Palliative RT can eliminate the signs of the compression of the oesophagus or superior caval vein; the usual dose is 10 × 3 Gy, using two opposed fields (recommendation category: 2b) (129).

#### Skin Metastases

Irradiation should be planned in line with the extent of the disease and the number and size of the foci (recommendation category: 2b) (129).

#### Intraocular and Orbital Metastasis

CT-based (if possible, 3D conformal) RT planning is applied and the dose is usually 10 × 3 Gy (recommendation category: 2b) (129).

This is part 2 of a series of 6 publications on the 1st Central-Eastern European Professional Consensus Statements on Breast Cancer covering imaging diagnosis and screening (131), pathological diagnosis (132), surgical treatment (133), systemic treatment (134), radiotherapy (present paper) of the disease and related follow-up, rehabilitation and psycho-oncological issues (135).

## Author’s Note

The consensus document contains product placement without the intention of advertising. Each complex molecular test is unique, and although these can be described without indicating their name (for example with the number of genes tested), not everyone will necessarily understand what this refers to. For this reason, and adopting the practice used in some of the source works, the tests are listed under their trade name.
